# A novel indicator for predicting functional recovery in elderly hip fracture patients

**DOI:** 10.3389/fmed.2025.1538038

**Published:** 2025-03-13

**Authors:** Weicheng Wu, Zhening Guo, Peiyao Zhu, Bo Lv, Yongtao Mao, Chang She, Wei Xu, Jun Gu, Jie Pan, Liubing Li

**Affiliations:** ^1^Department of Orthopedics, The Second Affiliated Hospital of Soochow University, Suzhou, China; ^2^Department of Pharmacy, The Second Affiliated Hospital of Soochow University, Suzhou, China; ^3^State Key Laboratory of Radiation Medicine and Protection, Soochow University, Suzhou, China

**Keywords:** hip fracture, HHS, LRCa^3^, inflammatory indices, functional recovery

## Abstract

**Background:**

The inflammatory response following hip fracture significantly influences postoperative functional recovery in patients. However, to date, no inflammatory index has been identified as a reliable and accurate predictor of functional recovery, especially in elderly patients with hip fractures. This study introduces and evaluates a novel inflammatory marker, the lymphocyte ratio-calcium index (LRCa^3^), for predicting one-year postoperative functional recovery and compares its performance to that of established markers, including the platelet-to-lymphocyte ratio (PLR), monocyte-to-lymphocyte ratio (MLR), systemic immune-inflammation index (SII), aggregate index of systemic inflammation (AISI), and systemic inflammation response index (SIRI).

**Methods:**

A retrospective analysis was conducted on 111 elderly patients (≥65 years) who underwent hip fracture surgery, and their demographic and laboratory data were analyzed. Patients were classified into good or poor recovery groups based on the Harris hip score (HHS) 1 year postoperatively. LRCa^3^ was calculated as the lymphocyte ratio multiplied by the cube of the serum calcium level. Logistic regression and receiver operating characteristic (ROC) curve analyses were performed to assess the predictive performance of the LRCa^3^ and other inflammatory indices. A nomogram prediction model was constructed.

**Results:**

ROC curve analysis revealed that, compared with the SII (AUC: 0.601), the SIRI (AUC: 0.61), the AISI (AUC: 0.577), and the MLR (AUC: 0.626) had superior predictive performance. Multivariate logistic regression revealed that the LRCa^3^ was an independent predictor of one-year functional recovery. The incorporation of LRCa^3^ into a nomogram further enhanced its predictive capacity, providing a more accurate tool for postoperative outcome assessment.

**Conclusion:**

LRCa^3^ is a novel and effective biomarker for predicting postoperative functional recovery in elderly hip fracture patients. Its integration into clinical practice could facilitate individualized patient management and improve long-term outcomes.

## 1 Introduction

With global population growth and the aging phenomenon, the incidence of osteoporotic fractures has been steadily increasing. Among these, hip fractures are the most severe because they are associated with high healthcare costs, elevated mortality rates, significant disability, and frequent complications. Consequently, hip fractures have emerged as a major global public health concern (1, 2). Projections indicate that by 2050, the global incidence of hip fractures will range between 6.26 million and 21.3 million cases (3, 4). Timely recovery of motor function after hip fracture surgery plays a critical role in reducing complications such as pneumonia and pressure ulcers. Furthermore, motor function recovery is strongly correlated with patient satisfaction with surgical outcomes. Despite this, research has shown that up to 80% of hip fracture patients continue to rely on assistive devices for daily activities 1 year postfracture (5). Therefore, enhancing postoperative motor function recovery in this population remains a pressing challenge in orthopedic and rehabilitative care.

Elderly patients with hip fractures often experience elevated release of inflammatory cytokines following injury because of reduced physiological reserves and insufficient anti-inflammatory mediators to maintain homeostasis. This heightened susceptibility to an exaggerated inflammatory response frequently leads to poorer prognoses for these patients (6). The inflammatory response has been proven to have a profound association with the adverse outcomes commonly observed in hip fracture patients. After a fracture, systemic immune-inflammatory cells such as neutrophils, monocytes, lymphocytes, and C-reactive protein (CRP) are secreted in large quantities. These cells produce cytokines such as IFN-*γ*, IL-6, IL-12, and TNF, which regulate adaptive immune responses and exhibit strong proinflammatory and antimicrobial effects (7, 8). Recent studies have identified IL-6, IL-8, CRP, and C1q as independent risk factors for poor postoperative joint function in hip fracture patients (9). Preoperative immune-inflammatory indicators, such as the neutrophil-to-lymphocyte ratio (NLR), platelet-to-lymphocyte ratio (PLR), monocyte-to-lymphocyte ratio (MLR), and CRP, are associated with increased all-cause mortality after surgery in elderly hip fracture patients (10, 11). The admission CAR (C-reactive protein-to-albumin ratio) and NLR have been shown to predict long-term mortality in elderly patients undergoing hip fracture surgery (7). Furthermore, the systemic immune-inflammation index (SII) on the fifth postoperative day can predict one-year mortality in hip fracture patients (12).

In recent years, inflammatory indices such as the MLR, PLR, SII, aggregate index of systemic inflammation (AISI), and systemic inflammation response index (SIRI) have been widely utilized across various diseases (13, 14). These indices have shown greater utility in assessing inflammation and predicting postoperative outcomes than single blood-based inflammatory markers do. However, their application is often limited by insufficient specificity and sensitivity, with inconsistent performance observed across different surgical procedures and patient populations. These limitations, coupled with contradictory statistical findings, underscore the need for further investigation to establish their reliability and applicability. Moreover, recent studies have highlighted several limitations of these indices in specific contexts. For example, the MLR and PLR have been reported to have no significant associations with postoperative mortality in hip fracture patients (15, 16). Similarly, the SII has been found to be inadequate for predicting the risk of spinal fractures (17). PLR is susceptible to fluctuations caused by platelet count variability resulting from surgical trauma. Although CRP is effective in identifying early postoperative inflammatory responses (18), it lacks the sensitivity and specificity necessary for evaluating long-term recovery (16). Research on the relationship between systemic immune-inflammatory responses and postoperative functional recovery in hip fracture patients is still limited, and no universally recognized predictive marker currently exists. This underscores the pressing need for novel biomarkers or indices to increase the accuracy of postoperative functional recovery predictions. One potential solution lies in combining inflammatory markers with bone metabolism-related indices, such as serum calcium levels. To address this, our study introduces a novel measurement index—the lymphocyte ratio-calcium index (LRCa^3^)—which is calculated by multiplying the lymphocyte ratio by the cube of the serum calcium level. The objective of this research was to evaluate whether the LRCa^3^ can effectively predict one-year postoperative motor function recovery in elderly hip fracture patients and to compare its predictive accuracy with that of commonly used immune-inflammatory indices. Ultimately, this study aims to provide clinicians with a more reliable and timelier tool for assessing inflammation severity in hip fracture patients, facilitating targeted interventions to improve outcomes.

## 2 Materials and methods

### 2.1 Study design

This retrospective study analyzed data from patients who underwent surgery for hip fractures at the Second Affiliated Hospital of Soochow University between March 2021 and March 2023. Eligible participants were aged 65 years or older and had undergone either hip replacement surgery or internal fixation. The exclusion criteria were as follows: patients with multiple injuries, ischemic necrosis, pathological fractures, previous hip surgeries, or incomplete medical records. To ensure that the focus remained on inflammatory biomarkers, we also excluded patients whose conditions, such as active infections, hematological disorders, or malignancies, could significantly alter their inflammatory status. The study adhered to the principles of the Declaration of Helsinki and received approval from the Ethics Committee of the Second Affiliated Hospital of Soochow University (JD-HG-2023-07).

### 2.2 Data collection

Medical records, including sex, age, fracture type, surgical procedure, and laboratory values measured on the first postoperative day, were reviewed to collect demographic and clinicopathological data. The laboratory parameters included the neutrophil ratio, white blood cell count, hemoglobin level, lymphocyte count, lymphocyte ratio, neutrophil count, monocyte count, platelet count, and serum calcium. The following inflammatory indices were calculated: (1) MLR = monocyte count (×10^9^/L)/lymphocyte count (×10^9^/L); (2) PLR = platelet count (×10^9^/L)/lymphocyte count (×10^9^/L); (3) SII = [platelet count (×10^9^/L) × neutrophil count (×10^9^/L)]/lymphocyte count (×10^9^/L); (4) AISI = neutrophil count (×10^9^/L) × platelet count (×10^9^/L) × monocyte count (×10^9^/L)/lymphocyte count (×10^9^/L); and (5) SIRI = neutrophil count (×10^9^/L) × monocyte count (×10^9^/L)/lymphocyte count (×10^9^/L). Postoperative functional recovery at 1 year was evaluated via the Harris hip score (HHS), a validated tool for assessing hip function, with a maximum score of 100. The HHS emphasizes functional aspects, including activities of daily living and gait (47 points) and pain (44 points), while assigning less weight to deformity (4 points) and range of motion (5 points) (5). Body mass index (BMI) was calculated as weight (kg) divided by height squared (m^2^). Patients were classified into two groups on the basis of their HHS: the good recovery group (≥80 points) and the poor recovery group (<80 points).

### 2.3 Statistical analysis

All the statistical analyses were performed via R software (version 4.0.2). Quantitative variables are expressed as the means ± standard deviations (SDs), whereas categorical variables are reported as counts with percentages. Group comparisons were conducted via the chi-square test for categorical data, the *t* test for variables with a normal distribution, and the Wilcoxon rank-sum test for variables with a nonnormal distribution. The predictive performance of inflammatory indices, including the SII, SIRI, AISI, PLR, and MLR, was assessed via receiver operating characteristic (ROC) curves. Logistic regression analyses were applied to develop both univariable and multivariable nutritional screening models. A nomogram prediction model was subsequently constructed on the basis of the regression results to estimate one-year postoperative functional recovery in elderly patients with hip fractures. The model’s discriminatory ability was evaluated by generating ROC curves and calculating the area under the curve (AUC). Finally, decision curve analysis (DCA) was performed to determine the clinical utility of the nomogram. A *p* value of <0.05 was considered to indicate statistical significance throughout the analysis.

## 3 Results

### 3.1 Clinicopathological characteristics

Within the primary cohort of 136 patients, after excluding those who were lost to follow-up, died, or had incomplete data, a total of 111 patients met the inclusion criteria and were enrolled in the study. Table 1 outlines the demographic characteristics of these patients. Among the participants, 28 (25.2%) were male, and 83 (74.8%) were female. Femoral neck fractures were observed in 67 patients (60.3%), whereas 44 patients (39.7%) had intertrochanteric fractures. At the one-year follow-up, postoperative hip function was classified as good in 60 patients and poor in 51 patients. Statistical analysis revealed significant differences in weight and BMI between the two groups (*p* < 0.05). However, no statistically significant differences were found in other variables, including sex, age, fracture type, serum hemoglobin, neutrophil count, lymphocyte count, or calcium level (*p* > 0.05).

**Table 1:** Comparison of clinical features between good recovery and poor recovery patients.

Variable	Harris<80	Harris> = 80	*p* value
*n*	51	60	
Gender (%)			
Female	42 (82.4)	41 (68.3)	0.14
Male	9 (17.6)	19 (31.7)	
Age	75.00 [69.00, 79.00]	80.50 [73.00, 85.25]	0.001
Height	1.57 [1.54, 1.64]	1.58 [1.54, 1.65]	0.924
Weight	60.00 [55.00, 65.00]	51.00 [46.00, 58.25]	<0.001
BMI	23.61 [22.22, 25.39]	20.25 [18.37, 23.23]	<0.001
Type of fraction (%)			
Femoral neck fracture	33 (64.7)	34 (56.7)	0.504
Intertrochanteric fracture	18 (35.3)	26 (43.3)	
Neutrophil ratio	82.20 [77.70, 85.05]	82.90 [81.12, 86.30]	0.028
White blood cell	8.70 [6.70, 10.60]	8.70 [7.27, 11.40]	0.288
Hemoglobin	98.00 [86.50, 112.00]	97.50 [88.50, 107.25]	0.707
Lymphocyte count	0.90 [0.65, 1.10]	0.70 [0.50, 0.83]	0.008
Lymphocyte ratio	9.60 [7.65, 12.90]	7.85 [5.57, 10.53]	0.001
Neutrophil count	6.40 [5.60, 8.70]	7.20 [6.07, 9.62]	0.191
Platelet	170.00 [131.00, 219.50]	164.00 [136.25, 189.75]	0.392
Mononuclear cell count	0.60 [0.40, 0.80]	0.60 [0.40, 0.80]	0.914
Calcium	2.04 [1.98, 2.12]	2.00 [1.88, 2.07]	0.019

BMI, body mass index.

### 3.2 Comparison of motor functional recovery among patients with LRCa^3^, the SII, the SIRI, the AISI, the PLR, and the MLR

**Figure 1:**
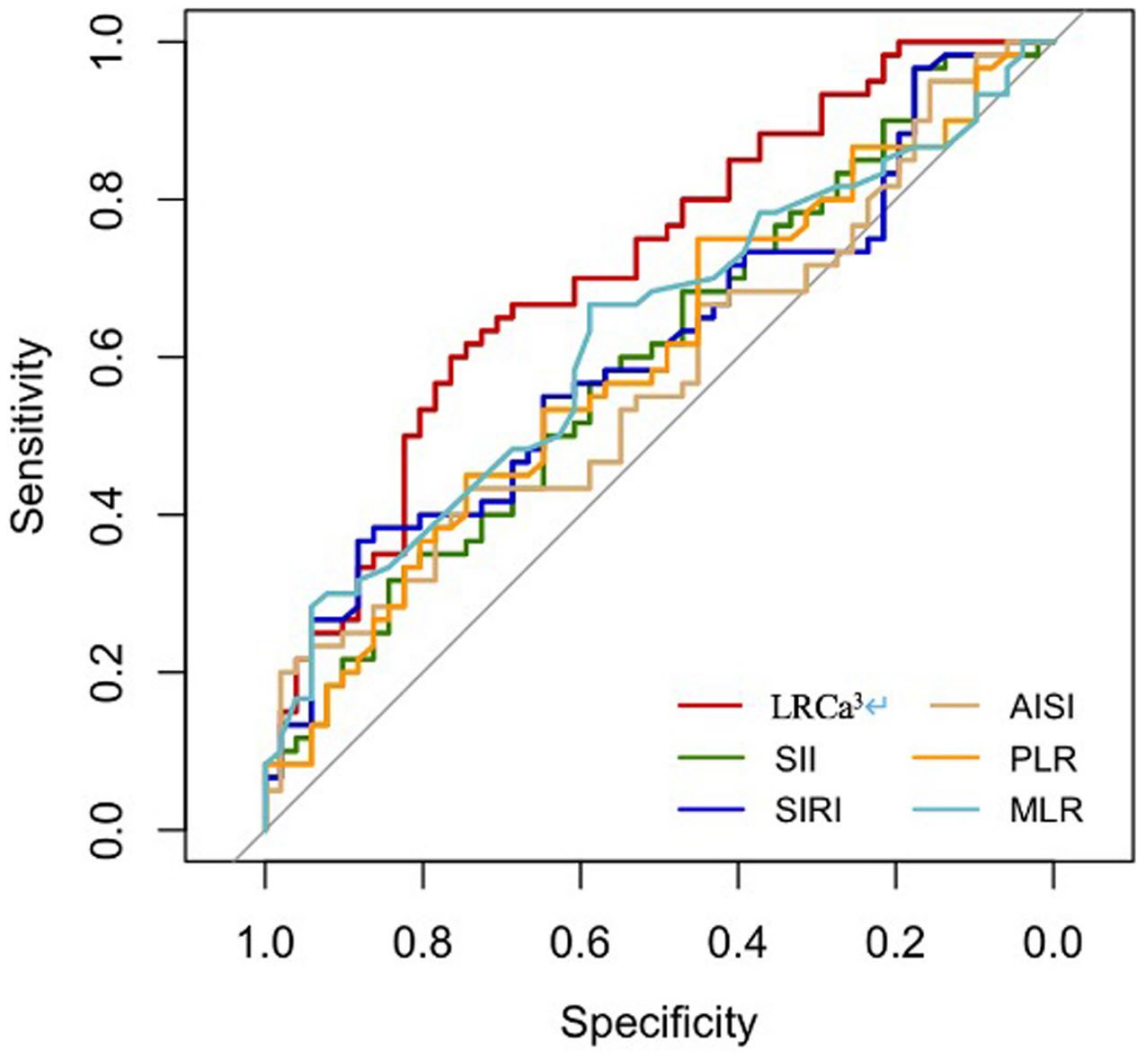
Receiver operating characteristic (ROC) curves of the lymphocyte ratio-calcium index (LRCa^³^), systemic immune-inflammation index (SII), systemic inflammation response index (SIRI), aggregate index of systemic inflammation (AISI), platelet-to-lymphocyte ratio (PLR), and monocyte-to-lymphocyte ratio (MLR) for predicting 1-year motor functional recovery.

The performance of the novel indicator LRCa^3^ was evaluated and compared with that of existing inflammatory indices (SII, SIRI, AISI, PLR, and MLR) for the prediction of one-year postoperative functional recovery in hip fracture patients via receiver operating characteristic (ROC) curves. As illustrated in Figure 1 and Table 2, the area under the curve (AUC) for LRCa^3^ was 0.715 (95% CI: 0.619–0.81; *p* < 0.05), surpassing the AUCs of the SII (0.601; 95% CI: 0.495–0.706; *p* < 0.05), SIRI (0.61; 95% CI: 0.504–0.715; *p* < 0.05), AISI (0.577; 95% CI: 0.471–0.684; *p* < 0.05), PLR (0.598; 95% CI: 0.492–0.704; *p* < 0.05), and MLR (0.626; 95% CI: 0.523–0.73; *p* < 0.05). These results underscore the superior predictive ability of LRCa^3^ compared with other inflammatory markers, highlighting its potential as a reliable tool for clinical assessment. Based on the ROC curve (Figure 1), the optimal cut-off for LRCa^3^ was determined to be 65.988, sensitivity of 63.5%, and specificity of 75.5%.

**Table 2:** Comparison between LRCa^3^ and other inflammation complex indices.

Indices	AUC [95% CI]	*p* values
LRCa^3^	0.715 [0.619, 0.81]	–
SII	0.601 [0.495, 0.706]	0.0242
SIRI	0.61 [0.504, 0.715]	0.0240
AISI	0.577 [0.471, 0.684]	0.0091
PLR	0.598 [0.492, 0.704]	0.0279
MLR	0.626 [0.523, 0.73]	0.0422

LRCa^3^, lymphocyte ratio-calcium index; SII, systemic immune-inflammation index; SIRI, systemic inflammation response index; AISI, aggregate index of systemic inflammation; PLR, platelet-to-lymphocyte ratio; MLR, monocyte-to-lymphocyte ratio.

### 3.3 Univariate and multivariate logistic regression analyses of the risk factors associated with HHS

As shown in Table 3, the lymphocyte ratio and calcium levels were combined to calculate LRCa3 for analysis. Univariate regression analysis revealed that age (OR: 1.1; 95% CI: 1.042–1.166; *p* = 0.001), BMI (OR: 0.8; 95% CI: 0.704–0.898; *p* < 0.001), the neutrophil ratio (OR: 1.134; 95% CI: 1.043–1.245; *p* = 0.005), and LRCa^3^ (OR: 0.975; 95% CI: 0.962–0.988; *p* < 0.001) were significantly associated with one-year postoperative functional recovery in hip fracture patients. After adjusting for covariates, multivariate regression analysis revealed BMI (OR: 0.845; 95% CI: 0.729–0.969; *p* = 0.019) and LRCa^3^ (OR: 0.979; 95% CI: 0.959–0.997; *p* < 0.001) as independent risk factors for poor functional recovery. These results highlight the predictive value of the novel indicator LRCa^3^, emphasizing its effectiveness in assessing postoperative functional outcomes in hip fracture patients.

**Table 3:** Univariate and multivariate logistic regression models.

	Univariate		Multivariate	
Variables	OR [95% CI]	*p* value	OR [95% CI]	*p* value
Gender (%)	2.163 [0.896, 5.537]	0.094		
Age	1.100 [1.042, 1.166]	0.001	1.062 [0.998, 1.134]	0.06
BMI	0.800 [0.704, 0.898]	<0.001	0.845 [0.729, 0.969]	0.019
Type of fraction (%)	1.402 [0.653, 3.052]	0.389		
Neutrophil rate	1.134 [1.043, 1.245]	0.005	0.976 [0.845, 1.126]	0.739
White blood cell	1.070 [0.933, 1.234]	0.336		
Hemoglobin	0.994 [0.970, 1.017]	0.589		
Lymphocyte count	0.162 [0.041, 0.573]	0.006		
Neutrophil count	1.110 [0.961, 1.292]	0.164		
Platelet	0.997 [0.991, 1.003]	0.373		
Mononuclear cell count	1.393 [0.336, 6.039]	0.649		
Calcium	0.034 [0.001, 0.684]	0.032		
LRCa^3^	0.976 [0.962, 0.988]	<0.001	0.979 [0.959, 0.997]	0.025

BMI, body mass index.

### 3.4 Validation of the predictive accuracy of the nomogram

**Figure 2:**
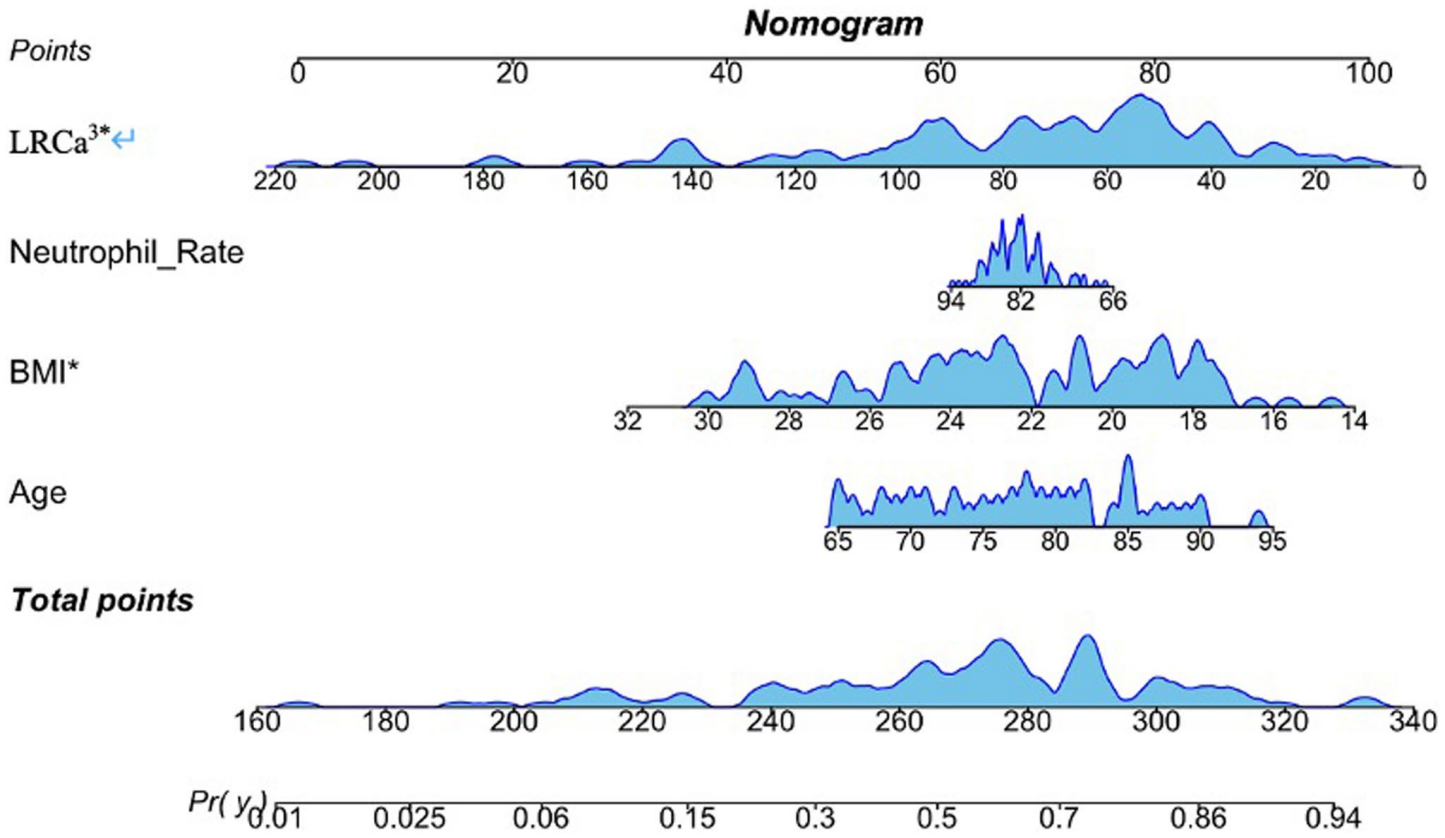
Nomogram model for predicting motor function prognosis. Age, neutrophil rate, body mass index (BMI) and the lymphocyte ratio-calcium index (LRCa^³^) were included.

**Figure 3:**
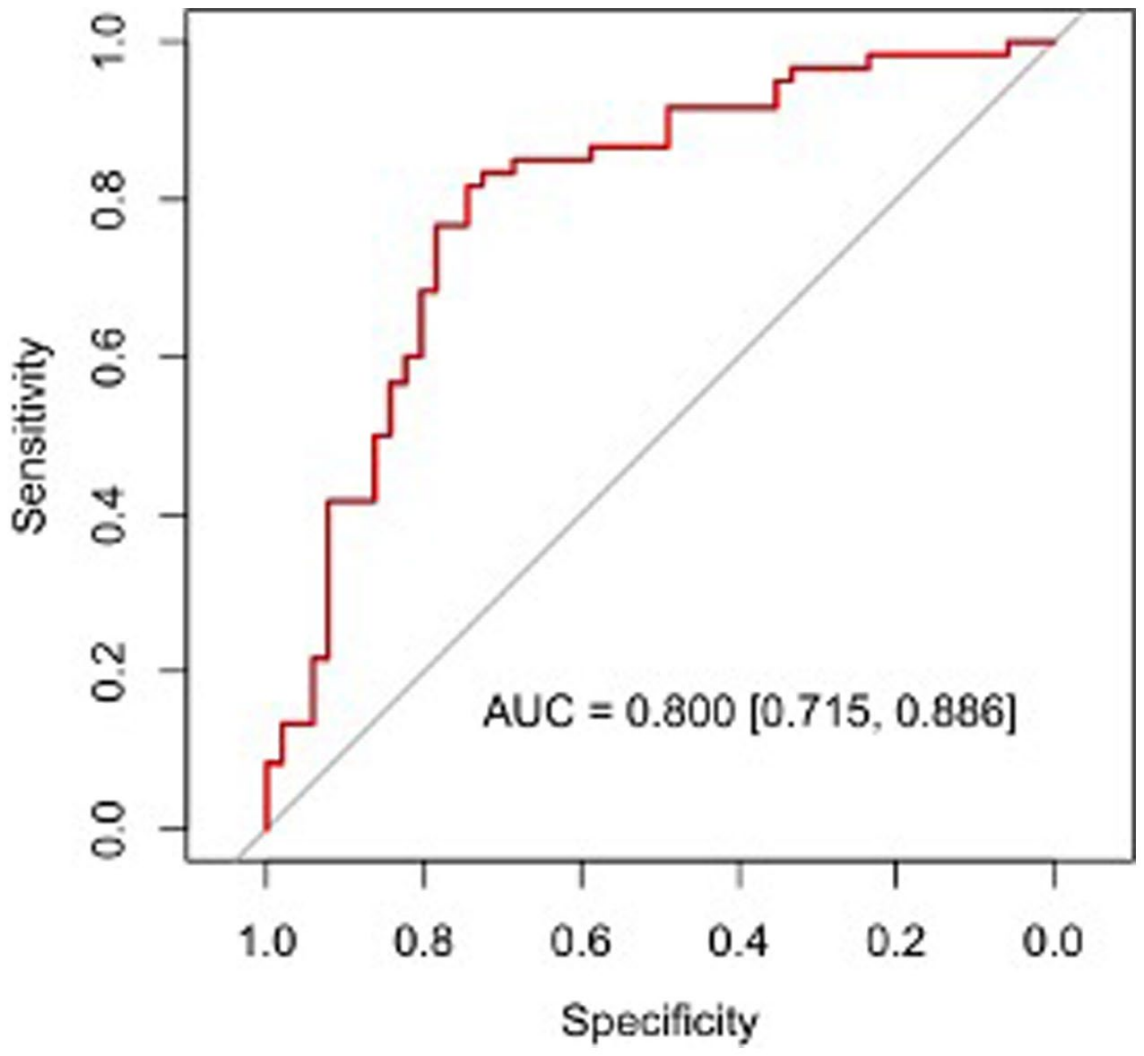
Receiver operating characteristic curves of the nomogram prediction model.

**Figure 4:**
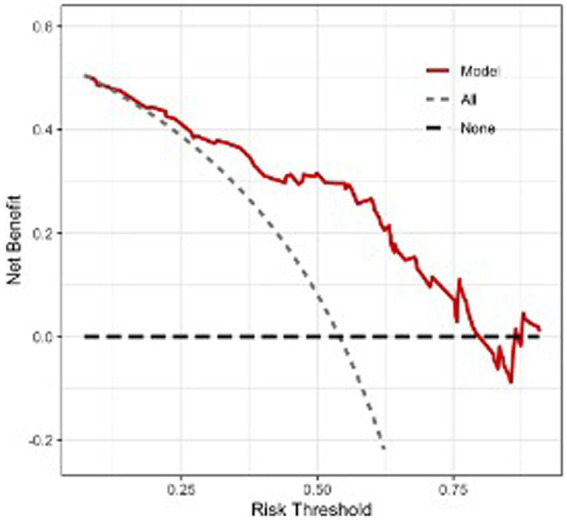
Decision curve analysis of the nomogram prediction model.

Regression analysis revealed that age, BMI, the neutrophil ratio, and LRCa^3^ were significant predictors of postoperative functional recovery. These variables were used to construct a nomogram prediction model, as shown in Figure 2. The model’s predictive performance was evaluated via a receiver operating characteristic (ROC) curve, which yielded an AUC of 0.800 (95% CI: 0.715–0.886), indicating strong predictive accuracy (Figure 3). Figure 4 presents the decision curve analysis (DCA) for the nomogram, demonstrating its clinical utility in predicting postoperative functional outcomes in elderly hip fracture patients. The DCA results suggest that clinical decisions guided by the nomogram can more effectively and accurately predict functional recovery following surgery.

## 4 Discussion

The most significant finding of this study is the identification of a novel inflammatory marker, LRCa^3^, which is derived from the lymphocyte ratio and serum calcium levels on postoperative day one. LRCa^3^ was shown to effectively predict one-year functional recovery in elderly patients undergoing hip fracture surgery. While previous studies have highlighted the prognostic value of inflammatory indices such as the AISI, PLR, SII, and SIRI in hip fracture patients, our ROC curve analysis demonstrated that LRCa^3^ offers superior sensitivity and specificity, establishing it as a more reliable predictor of postoperative functional recovery, and we determined the optimal cut-off value to be 65.988. Unlike traditional inflammatory indices, which focus solely on inflammation, LRCa^3^ integrates immune function with bone metabolism. This broader scope likely contributes to its enhanced predictive performance. By combining the lymphocyte ratio and calcium level as a composite marker, LRCa^3^ provides a novel approach for predicting functional outcomes in elderly hip fracture patients, a relationship not previously explored in the literature. These findings underscore the potential of LRCa3 as a valuable tool in clinical practice for tailoring postoperative management strategies.

Systemic immune and inflammatory responses play critical roles in determining postoperative outcomes in hip fracture patients, with various inflammatory mediators contributing significantly to fracture-related trauma and bone healing processes (19). The lymphocyte ratio, an indicator of immune status, has been linked to the risk of postoperative complications and infections. Neutrophils, the primary effector cells of the innate immune response, function in tandem with lymphocytes, which regulate immune system activities (8, 20). Platelets, among the first responders to initiate the inflammatory cascade, work alongside monocytes and macrophages, which possess strong phagocytic capabilities. These latter cells are recruited shortly after neutrophils and persist in sites of chronic inflammation and infection, where they release cytokines that modulate adaptive immune responses. Traditional inflammatory indices are typically derived from ratios of two or three immune pathways to quantify inflammation intensity. While these indices, such as the AISI, PLR, SII, and SIRI, have demonstrated robust predictive power in conditions such as metabolic disorders, cardiovascular diseases, and cancer (21, 22), their applicability to elderly hip fracture patients is less certain. These indices may fail to adequately reflect the intricate interplay of immune and inflammatory processes unique to this patient population, underscoring the need for more comprehensive predictive tools tailored to their specific physiological and pathological characteristics.

Serum calcium levels serve as indicators of both bone metabolic status and mineral balance. Dysregulation of calcium metabolism is often associated with osteoporosis and delayed fracture healing. In addition to its structural role, calcium also contributes to inflammatory responses by regulating various cellular functions through calcium signaling pathways, including immune cell activation and the release of inflammatory mediators (23). Gordon et al. reported that hypercalcemia is linked to elevated inflammatory markers, reduced bone density, and the modulation of chemokine production by extracellular calcium, which acts as a strong regulator of immune cell activity (24). Rossol et al. demonstrated that extracellular calcium stimulates the NLRP3 inflammasome, prompting monocytes and macrophages in the innate immune system to produce IL-1 (25). Similarly, Zhu et al. reported that reducing calcium levels inhibits NLRP3 activity, thereby decreasing IL-1β production via this pathway (26). Li et al. reported a U-shaped relationship between inflammatory markers and vitamin D levels (25 (OH) D and 1,25 (OH)D), with serum calcium positively correlated with both forms of vitamin D. However, when calcium levels exceed a certain threshold, they become positively associated with inflammatory markers (27). Research has further suggested that bone destruction and calcium release driven by inflammation can increase serum calcium levels and decrease bone density. This phenomenon is evident in rheumatoid arthritis patients, where approximately 30% exhibit hypercalcemia (24, 28). Serum calcium inherently reflects the dynamic interplay between osteoblast and osteoclast activity, making it a critical marker of bone health. For elderly patients with fractures, close monitoring of calcium level fluctuations is essential for understanding their bone metabolism and guiding effective recovery strategies.

Bone loss is intricately linked to inflammation and the immune system, with the skeletal system being highly responsive to chronic inflammatory stress. Conditions such as osteoarthritis and gout are frequently associated with osteoporosis, highlighting the interplay between chronic inflammation and bone health (29, 30). Inflammatory responses in fracture patients accelerate bone destruction, leading to elevated serum calcium levels. These increased calcium levels further stimulate the production of chemokines by immune cells, perpetuating a cycle of inflammation and bone degradation. This bidirectional relationship underscores the advantage of combining calcium levels with inflammatory markers, such as the lymphocyte ratio, to improve the accuracy of postoperative functional recovery predictions in elderly hip fracture patients. The introduction of the LRCa^3^ index offers clinicians a valuable tool for identifying high-risk patients during the preoperative or early postoperative phases. This allows for the implementation of tailored management strategies. Patients with compromised immune function and disrupted calcium metabolism may benefit from extended rehabilitation programs or nutritional supplementation to optimize functional recovery after surgery.

This study has certain limitations. The relatively small sample size and single-center design may limit the generalizability of the findings. To strengthen the validity and applicability of the results, future studies involving larger sample sizes and multicenter designs are necessary to further evaluate the predictive efficacy of LRCa^3^. Moreover, this study focused exclusively on one-year postoperative functional recovery in elderly hip fracture patients. Future research should consider extending the follow-up period to assess long-term functional recovery and quality of life, providing a more comprehensive understanding of patient outcomes over time.

## 5 Conclusion

This study identified a novel specific indicator, LRCa^3^, measured on postoperative day one, which effectively predicts one-year functional recovery in elderly hip fracture patients. Compared with commonly used inflammatory indices, LRCa^3^ has superior predictive value. Using this indicator, a predictive model was developed and validated, confirming its efficacy. This model provides significant clinical value by assisting in preoperative assessment and guiding postoperative treatment decisions, thereby enhancing management strategies for elderly hip fracture patients.

## Data Availability

The raw data supporting the conclusions of this article will be made available by the authors, without undue reservation.
